# Report of a Case of Poisoning by Corrosive Sublimate

**Published:** 1858-09

**Authors:** John Boardman


### ART. II.—*Report of Cases of Poisoning by Corrosive Sublimate.* By John Boardman, M. D.

Perhaps the following cases of poisoning by corrosive sublimate may interest, although the description of the first is not perfect, the notes which I made from day to day having been lost:

Case I. April 29, 1856. M. B., Irish, aged 21 years, about 1, P. M., while assisting a family to move, thinking a bottle contained brandy, swallowed one mouthful of the contents, but spit out the second because the first burnt his mouth and throat. In about twenty minutes he learned from the lady of the house that he had taken "bed-bug poison." In the meantime he had thrown off a portion of his dinner, by irritating the fauces with his fingers. He immediately came to my office, but I not being in, nearly one hour passed before he called upon Dr. Newman, who produced free and copious emesis by the use of zinci sulph., administering to him from time to time the white of eggs. He remained in Dr. N.'s office for about one and a-half hours, and then left for home.

I saw the patient about 7, P. M., and found that he had vomited three or four times on his way home; also his bowels had moved three times that afternoon, and that the family had called a German doctor, who, at 5, P. M., gave him a large dose of salts and senna which had not operated.

The patient appeared very comfortable, although anxious and exhausted, which I attributed to the fright and the copious operation of the emetic. The pulse was rather feeble, about 100 per minute; no tenderness over the abdomen on pressure; mouth and fauces looked as if they had been seared. He had some thirst and complained of a slight soreness in the throat, which he located opposite the sup. ext. of the sternum.

I ordered milk, also an infusion of ulmi cortex, to be used as drinks, and that no other nourishment should be given till I saw him.

April 30. This A. M. I found that the salts had operated very freely last evening. He had passed a quiet night. There was increased thirst, and the soreness of the throat was more marked; the back part of the mouth was a little swollen; pulse soft, about 75; no tenderness of the abdomen. He seemed doing so well, that ordering him to use nothing but the milk and ulmi cortex, I left him for a few days.

May 3. Was called this A. M. to see him. Found patient with a slight fever; pulse about 84, a little harder than natural; tongue thickly coated with a white fur; back of mouth swollen; deglutition painful; stomach irritable; bowels costive; no pain or tenderness in the abdomen; had been troubled a little with hiccough. The patient had passed but a few drops of urine since the 29th, and this was the reason of sending for me. He had remained in bed, but had not suffered except from his throat, since I was there. The bladder was empty and I could not discover any trouble from suppression.

I continued his drinks and ordered spr. nit. dulce, ʒj. every 4 hours, also pulv. opii, gr. ss., if the hiccough returned.

May 4. Has not passed any water; pulse more frequent and rather feeble; mouth and throat much swollen and painful; stomach quite irritable, could not retain the spir. nit.

May 5, A. M. Has not passed any water. Had six watery movements of the bowels during the night; stomach irritable; appeared quite weak; deglutition quite difficult.

Sulph. morph., gr. $\frac{1}{6}$, at 4 hours.

5, P. M. Patient's general condition much the same. Has passed no water; bowels had not moved since morning; slight tenderness over the abdomen on pressure. I passed a catheter into the bladder but found it quite empty.

Sulph. morph., gr. $\frac{1}{6}$, at 6 hours; also port wine, ʒij, at 6 hours.

May 6th. Has not passed any water; complains much of his mouth, throat, and of the difficulty of swallowing; has spit up some blood; no pain or tenderness of the abdomen; pulse about 130, feeble; complains of a sense of general prostration; bowels have moved two or three times since last night. The wine burnt his throat even when diluted.

Suspend wine; continue sulph. morph.; give freely of beef essence.

May 7th. Patient died at 8, A. M., while sitting on the vessel, quite unexpectedly to his friends, who had not seen any particular change in his condition for a number of hours.

Case II. April 7th, 1858. G., aged 22 years. About 9½, A. M., after a moderate breakfast, took a large swallow of a strong solution of corrosive sublimate with the intention of self-destruction, but suffering caused by the burning in the mouth and throat induced him to inform the family almost immediately of what he had done. He was given freely the white of eggs, also mustard and warm water, and Dr. Hamilton and myself were sent for.

We saw the patient together about 10, A. M. Then the pulse was about 90, irritable; countenance haggard and anxious. He complained of great pain in the stomach, also in the throat, which he located opposite the sup. ext. of the sternum, and of some difficulty in deglutition. Part of the time he was walking about the room, occasionally vomiting, and part of the time writhing as one with a severe attack of colic.

We continued the use of the white of eggs and warm water till the stomach appeared thoroughly washed out. Spr. rhei, ℥vi, was administered, but almost immediately rejected by the stomach, and a warm mustard poultice was applied to the abdomen. His bowels moved once moderately about 10½, A. M. Gave him milk and infusion of ulmi cortex as drinks.

His pain remained severe in the throat and abdomen, till about 12, M., from which time he gradually became more comfortable.

April 8th. Found the patient partly dressed. He had passed a quiet night and felt comparatively well. There was slight tenderness over the abdomen and a very little soreness in the throat.

Ordered him to remain quiet in bed, and to use nothing but the milk and ulmi cortex that day.

April 9th. Found patient still better; his mouth was slightly ptyalized. Let him get up.

He continued to improve, and in one week was as well as before.

I have given this account in the hope of hearing from others who may have met with like cases in their own practice, for I think but little is known as yet of the symptoms and treatment of such cases.

Death seemed in Case I, not as I had at first expected, to approach from inflammation of the throat and stomach, but from the effects of a peculiar poison. I did not find at any time, symptoms that called my attention to any organ, save the urinary, and the fault there seemed to be more a want of action than anything else. The general system did not seem to suffer from the suppression of urine. I know, that at the time, I was led to doubt whether the want of action of the kidneys might not be on account of not finding material to work upon.

